# Supplementary material for: Impact of Phyllosphere Methylobacterium on Host Rice Landraces
Source: Microbiol Spectr. 2022 Jul 20;10(4):e00810-22. doi: 10.1128/spectrum.00810-22 (PMC9431194; doi:10.1128/spectrum.00810-22)
Supplement: Supplemental file 2 — Tables S1 to S6 and Fig. S1 to S8. Download spectrum.00810-22-s0002.pdf, PDF file, 1.2 MB [file spectrum.00810-22-s0002.pdf]

**SUPPLEMENTARY TABLES**

**Table S1**: Nutrients received by each plant after foliar spraying of 10 ml broth (sterile media without bacteria).

| Components | Composition | g/mL | g/plant |
|---|---|---|---|
| Phosphate solution (P) | $K_2HPO_4$ | 0.00126 | 0.0126 |
| | $NaH_2PO_4$ | 0.001125 | 0.01125 |
| Sulphate solution (S) | $(NH_4)_2SO_4$ | 0.00025 | 0.0025 |
| | $MgSO_4.7H_2O$ | 0.0001 | 0.001 |
| Metal Mix | $ZnSO_4.7H_2O$ | 0.00018 | 0.0018 |
| | $CaCl_2.2H_2O$ | 0.00146 | 0.0146 |
| | $MnCl_2.2H_2O$ | 0.000107 | 0.00107 |
| | $FeSO_4.7H_2O$ | 0.0025 | 0.025 |
| | $(NH4)_6.Mo_7O_{24}.4H_2O$ | 0.00018 | 0.0018 |
| | $CuSO_4.5H_2O$ | 0.000187 | 0.00187 |
| Methanol (60 mM) | | | 0.6 mili moles |
| Succinate (2 mM) | | | 0.02 mili moles |

**Table S2**: Primers used for qRT-PCR for rice gene.

| Primer name | Sequence 5'-3' | Target gene | Ref |
|---|---|---|---|
| Xa21 F | CTCCCATTACTGCTCTTCGTCC | Xa21 receptor | (1) |
| Xa21 R | GACGTGTTCCAAGATGCCAGCG | | |
| OsFLS2 F | GTCGCAGCATTACACGAAGACG | Flagella receptor | (2) |
| OsFLS2 R | ACGCCGTTCTTGAACTCCAGC | | |
| OsPR1b F | CATGGTAGCCGCCATGGCAC | Pathogenesis-related gene | (3) |
| OsPR1b R | GTTGCTGGAGTGGATCAGGCTG | | |
| OsPR8 F | CATCGGCGCGCAGTTCACCGG | | |
| OsPR8 R | GCGAGGATGACGCTGAGGTTG | | |
| OsMPK3 F | CTCTCCCTCATCGCCTTGCTGTC | MAP kinases | This study |
| OsMPK3 R | CTGGTACTTGTTCGTCACCTCG | | |
| OsMPK6 F | GCATATGAATTGATGGATACTGATCTGC | | |
| OsMPK6 R | CTGAGGTGGTACGAGCAAGTCC | | |
| OsWRKY62 F | GCCGCTCTTCAGCCGATCG | WRKYs transcription factors | (4) |
| OsWRKY62 R | GGTGGGCGTGGTGACCGGC | | |
| OsWRKY13 F | TTTGGGAAAGCGTTGGATTAGT | | This study |
| OsWRKY13 R | GCGCACACACACTCCAACTC | | |
| OsWRKY76 F | CCTCGACCTCTGCGTCGGG | | |
| OsWRKY76 R | CTCATCTGAGTGACCTTCGC | | |
| OsWRKY53 F | CTTCGTCACGGTCTCCTCGCTTC | | |
| OsWRKY53 R | GCTGCGCGGACTTGAACTTG | | |
| OsACO1 F | CATCAACATGGAGTTGCTCGC | ACC-oxidase | (5) |
| OsACO1 R | CGCACACGCTTGTAGTGGTC | | |
| OsPAL1 F | CGGTCGTTCCCGCTCTAC | Phenylalanine ammonia-lyase gene | (6) |
| OsPAL1 R | TCGCCGTTCCACTCCTT | | |
| OsPAL4 F | GGAGGACCAAGGAGGGT | | |
| OsPAL4 R | GGAGTGTGTTGATGCGG | | |
| Osg1 F | GTATGGGACAAAGGATCTCC | Beta-glucanases | (5) |
| Osg1 R | GTGATCCAGCCCTACTGCTG | | |
| OsPR1#74 F | GTATGCTATGCTACGTGTTTATGC | Pathogenesis-related gene | (3) |
| OsPR1#74 R | GCAAATACGGCTGACAGTACAG | | |
| OsPR1#11 F | ACGCCTTCACGGTCCATAC | | |
| OsPR1#11 R | AAACAGAAAGAAACAGAGGGAGTAC | | |
| SNAC1 F | GGGTGCTGTGTCGGCTGTA | Transcription factor | (4) |
| SNAC1 R | CGACTGCGACGTAACCATGT | | |

**Table S3**: Pairwise comparisons across treatments, and treatment x landrace interaction effects on plant traits. Significant P-values are represented in bold font.

| **A. Treatment: Untreated vs. treated** | | | | | | |
|---|---|---|---|---|---|---|
| **Model = ANOVA** | Treatment | | Landrace | | Landrace x Treatment | |
| Plant traits | F | P | F | P | F | P |
| Growth rate | 40.768 | <**0.001** | 202.685 | <**0.001** | 2.779 | **0.065** |
| Height | 33.344 | <**0.01** | 546.668 | <**0.001** | 4.193 | **0.0167** |
| Flag leaf length | 0.158 | **0.07** | 35.54 | 0.7 | 20.389 | <**0.001** |
| Flag leaf width | 28.843 | <**0.001** | 127.396 | <**0.001** | 3.587 | **0.03** |
| Number of tillers | 1.66 | 0.2 | 2.322 | 0.1 | 0.285 | 0.752 |
| Number of panicles | 12.6 | <**0.001** | 5.314 | **0.006** | 3.122 | **0.047** |
| Yield | 45.6 | <**0.001** | 13.42 | <**0.001** | 9.245 | **<0.001** |
| Grain weight | 1.06 | 0.3 | 2.43 | **0.09** | 0.415 | 0.7 |
| Filled grains | 13.46 | <**0.001** | 42.398 | <**0.001** | 4.733 | **<0.001** |
| **B. Treatment: Untreated vs. broth** | | | | | | |
| Growth-rate | 16.62 | <**0.001** | 82.03 | <**0.001** | 0.72 | 0.5 |
| Height | 14.4 | <**0.001** | 229.1 | <**0.001** | 0.65 | 0.5 |
| Flag leaf length | 0.7 | 0.4 | 4.18 | **0.01** | 13.4 | <**0.001** |
| Flag leaf width | 16.14 | <**0.001** | 79.4 | <**0.001** | 3.08 | **0.05** |
| Number of tillers | 0.058 | 0.8 | 0.44 | 0.6 | 1.5 | 0.2 |
| Number of panicles | 21.37 | <**0.001** | 1.89 | 0.15 | 5.44 | **0.006** |
| Yield | 53.22 | <**0.001** | 3.45 | **0.037** | 11.03 | <**0.001** |
| Grain weight | 2.33 | 0.13 | 3.14 | **0.049** | 1.14 | 0.3 |
| Filled grains | 49.56 | <**0.001** | 4.14 | **0.019** | 5.36 | **0.006** |
| **C. Treatment: Broth vs. own** | | | | | | |
| Growth-rate | 0.005 | 0.9 | 100.52 | <**0.001** | 0.077 | 0.9 |
| Height | 0.43 | 0.5 | 269.6 | <**0.001** | 0.668 | 0.5 |
| Flag leaf length | 9.14 | <**0.001** | 0.94 | 0.4 | 1.33 | 0.3 |
| Flag leaf width | 0.239 | 0.6 | 74.3 | <**0.001** | 2.13 | 0.12 |
| Number of tillers | 9.607 | <**0.001** | 1.6 | 0.2 | 1.7 | 0.2 |
| Number of panicles | 0.1 | 0.7 | 6.26 | **0.003** | 0.412 | 0.6 |
| Yield | 0.92 | 0.3 | 18 | <**0.001** | 0.99 | 0.4 |
| Grain weight | 0.3 | 0.6 | 1.77 | 0.2 | 0.46 | 0.6 |
| Filled grains | 0.52 | 0.5 | 10.7 | <**0.001** | 0.73 | 0.5 |
| **D. Treatment: Broth vs. other** | | | | | | |
| Growth-rate | 14.6 | <**0.001** | 131.88 | <**0.001** | 2.4 | 0.096 |
| Height | 16.1 | <**0.001** | 366.6 | <**0.001** | 5.39 | **<0.001** |
| Flag leaf length | 0.91 | 0.3 | 1.408 | 0.25 | 0.561 | 0.6 |
| Flag leaf width | 5.84 | **0.018** | 42.88 | <**0.001** | 0.71 | 0.50 |
| Number of tillers | 0.075 | 0.78 | 0.38 | 0.7 | 1.84 | 0.16 |

| | | | | | | |
|---|---|---|---|---|---|---|
| Number of panicles | 20.5 | <**0.001** | 2.628 | **0.078** | 3.4 | **0.04** |
| Yield | 18.6 | <**0.001** | 10.35 | **<0.001** | 2.55 | 0.084 |
| Grain weight | 1.81 | 0.18 | 4.55 | **0.014** | 0.46 | 0.6 |
| Filled grains | 14.14 | <**0.001** | 6.4 | <**0.001** | 1.03 | 0.36 |

**Table S4**: Pairwise comparisons across treatments on plant traits after removing the influential data points using GLM followed by Tukey's HSD. Significant P-values are represented in bold font.

| Landrace | Plant traits | Untreated - Broth only | Own-Broth only | Other-Broth only | Untreated-Own | Untreated -Other |
|---|---|---|---|---|---|---|
| Chakhao | Growth rate | **<0.001** | 0.97 | 0.99 | **0.00182** | **0.0014** |
| | Height | **0.05** | 0.77 | 0.75 | 0.38 | **0.002** |
| | Flag leaf length | **<0.001** | **0.001** | 0.87 | 0.14 | **< 0.001** |
| | Flag leaf width | **<0.001** | **0.07** | 0.71 | **0.08** | **<0.001** |
| | Number of tillers | 0.98 | 0.15 | 0.95 | 0.26 | 0.99 |
| | Number of panicles | 0.96 | 0.83 | 0.78 | 0.56 | 0.97 |
| | Yield | 0.52 | 0.35 | 0.53 | **0.016** | 1 |
| | Weight per grain | 0.12 | 0.29 | 1 | 0.94 | 0.11 |
| | Number of filled grains | 0.76 | **0.066** | 0.97 | **0.002** | 0.94 |
| | Percent filled grains | **0.007** | **0.003** | 0.77 | **< 0.001** | 0.095 |
| Phouren-mubi | Growth rate | 0.24 | 0.98 | 0.75 | 0.45 | **0.024** |
| | Height | 0.59 | 0.68 | 0.28 | 0.99 | **0.014** |
| | Flag leaf length | 0.73 | **0.001** | 0.68 | **0.026** | 0.99 |
| | Flag leaf width | **0.012** | 0.65 | 0.3 | **<0.001** | **<0.001** |
| | Number of tillers | 0.95 | **0.003** | 0.89 | **0.02** | 0.9 |
| | Number of panicles | **<0.001** | 0.98 | **<0.001** | **< 1e-05** | 0.91 |
| | Yield | **<0.001** | 0.99 | **<0.001** | **< 1e-04** | **0.0005** |
| | Weight per grain | **<0.001** | 0.78 | **<0.001** | 0.26 | 0.65 |
| | Number of filled grains | **<0.001** | 0.32 | **<0.001** | **< 1e-04** | **0.00014** |
| | Percent filled grains | **<0.001** | 0.36 | 0.68 | **<0.001** | **<0.001** |
| Phou-ngang | Growth rate | **<0.001** | 0.99 | **<0.001** | **0.00019** | **< 1e-04** |
| | Height | **<0.001** | 0.94 | **<0.001** | **< 0.001** | **< 0.001** |
| | Flag leaf length | **<0.001** | 0.56 | **0.02** | **<0.001** | 0.22 |
| | Flag leaf width | 0.72 | 1 | 0.94 | 0.72 | 0.36 |
| | Number of tillers | 0.98 | 0.5 | 0.93 | 0.262 | 0.99 |
| | Number of panicles | **0.088** | 0.85 | 0.1 | 0.4 | 0.99 |
| | Yield | **0.03** | 0.96 | 0.21 | **0.078** | 0.78 |
| | Weight per grain | 0.69 | 0.75 | 0.73 | 1 | 1 |
| | Number of filled grains | **<0.001** | 0.71 | **0.04** | **0.017** | 0.44 |
| | Percent filled grains | 0.94 | 1 | 0.45 | 0.96 | 0.78 |

**Table S5**: *Methylobacterium* isolates with distinct colony morphology, identified from leaf imprints, 2 months after foliar spray. Isolates were identified as "own" or "other" only when they showed 100% 16S rRNA sequence identity.

| **Landrace** | **Samples** | **Total *Methylobacterium* isolates** | **Number of own/other isolates** | **Number of environmental isolates** |
|---|---|---|---|---|
| Chakhao | Untreated | 2 | 0 | 2 |
| | Broth control | 5 | 2 (own) | 3 |
| | Own | 10 | 4 (own) | 6 |
| | Other | 9 | 0 | 9 |
| Phouren-mubi | Untreated | 6 | 0 | 6 |
| | Broth control | 3 | 1 (own) | 2 |
| | Own | 2 | 0 | 2 |
| | Other | 1 | 1 (own) | 0 |
| Phou-ngang | Untreated | 7 | 1 (own/other) | 6 |
| | Broth control | 1 | 0 | 1 |
| | Own | 5 | 0 | 5 |
| | Other | 0 | 0 | 0 |

**Table S6**: Model summary showing time and treatment effects on the expression of rice plant genes. Significant P-values are represented in bold font.

| **A. Time: Initial vs. 48 hpi** | | | | | | |
|---|---|---|---|---|---|---|
| **Model = ANOVA** | Time | | Treatment | | Time * Treatment | |
| Genes | F | P | F | P | F | P |
| XA21 | 1.08 | 0.3 | 0.05 | 0.98 | 0.05 | 0.98 |
| FLS2 | 3.17 | **0.04** | 0.49 | 0.69 | 0.19 | 0.99 |
| MAPK6 | 2.36 | 0.089 | 1.29 | 0.29 | 1.44 | 0.2 |
| WRKY13 | 3.79 | **0.07** | 0.088 | 0.96 | 0.088 | 0.96 |
| WRKY53 | 1.76 | 0.2 | 0.076 | 0.97 | 0.076 | 0.97 |
| SNAC1 | 4.61 | **0.047** | 0.311 | 0.81 | 0.311 | 0.81 |
| PR1#74 | 4.7 | **0.045** | 1.4 | 0.3 | 1.4 | 0.3 |
| OsPAL4 | 2.34 | 0.145 | 0.73 | 0.54 | 0.73 | 0.54 |
| OsACO1 | 4.92 | **0.04** | 1.12 | 0.37 | 1.12 | 0.37 |
| OsG1 | 0.6 | 0.45 | 3.7 | **0.05** | 3.7 | **0.05** |
| **B. Time: Before vs. after spraying** | | | | | | |
| XA21 | 0.9 | 0.35 | 0.37 | 0.77 | 0.744 | 0.5 |
| FLS2 | 1.57 | 0.23 | 0.66 | 0.6 | 0.35 | 0.8 |
| MAPK6 | 1.27 | 0.27 | 1.71 | 0.2 | 2.22 | 0.12 |
| WRKY13 | 0.688 | 0.419 | 0.39 | 0.76 | 0.97 | 0.43 |
| WRKY53 | 2.463 | 0.13 | 0.21 | 0.89 | 0.95 | 0.44 |
| SNAC1 | 3.546 | **0.07** | 3.08 | **0.06** | 2.75 | **0.077** |
| PR1#74 | 0.92 | 0.35 | 0.69 | 0.56 | 2.83 | **0.07** |
| OsPAL4 | 0.63 | 0.44 | 1.17 | 0.35 | 1.06 | 0.39 |
| OsACO1 | 0.46 | 0.5 | 0.44 | 0.72 | 0.138 | 0.94 |
| OsG1 | 1.85 | 0.19 | 0.47 | 0.7 | 1.2 | 0.34 |

## SUPPLEMENTARY FIGURES

**Fig. S1**: **Experimental field plot**. A. View of the experimental field plot before and after transplantation of the rice plants. B. Inoculum used for treating the seedlings and foliar spraying on the rice plants. C. Transplantation of rice seedlings with enough spacing using a wooden frame.

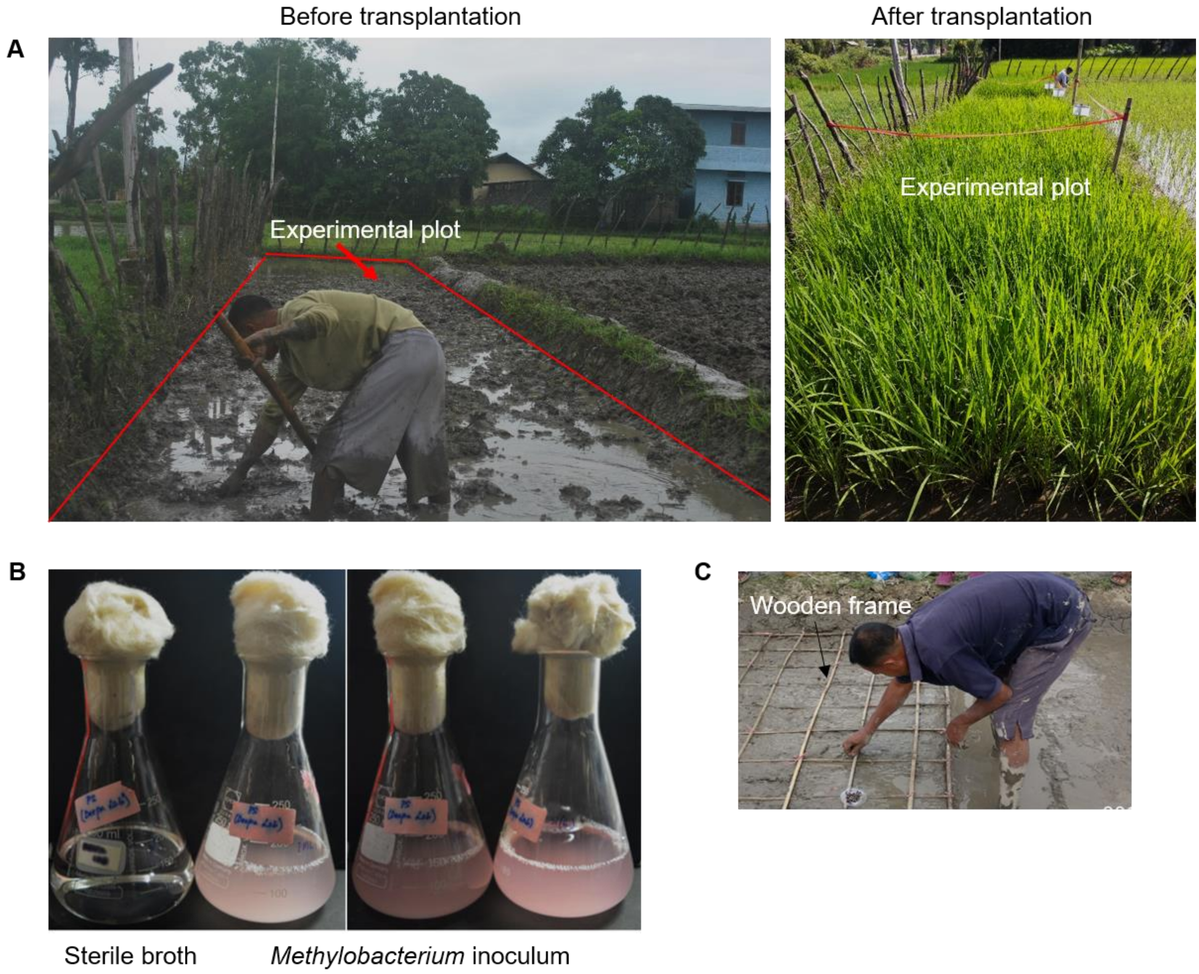

**Fig. S2**: **Experimental design of greenhouse experiment**. Schematic showing the timepoints for treatments and RNA extraction to analyze host gene expression after bacterial treatment.

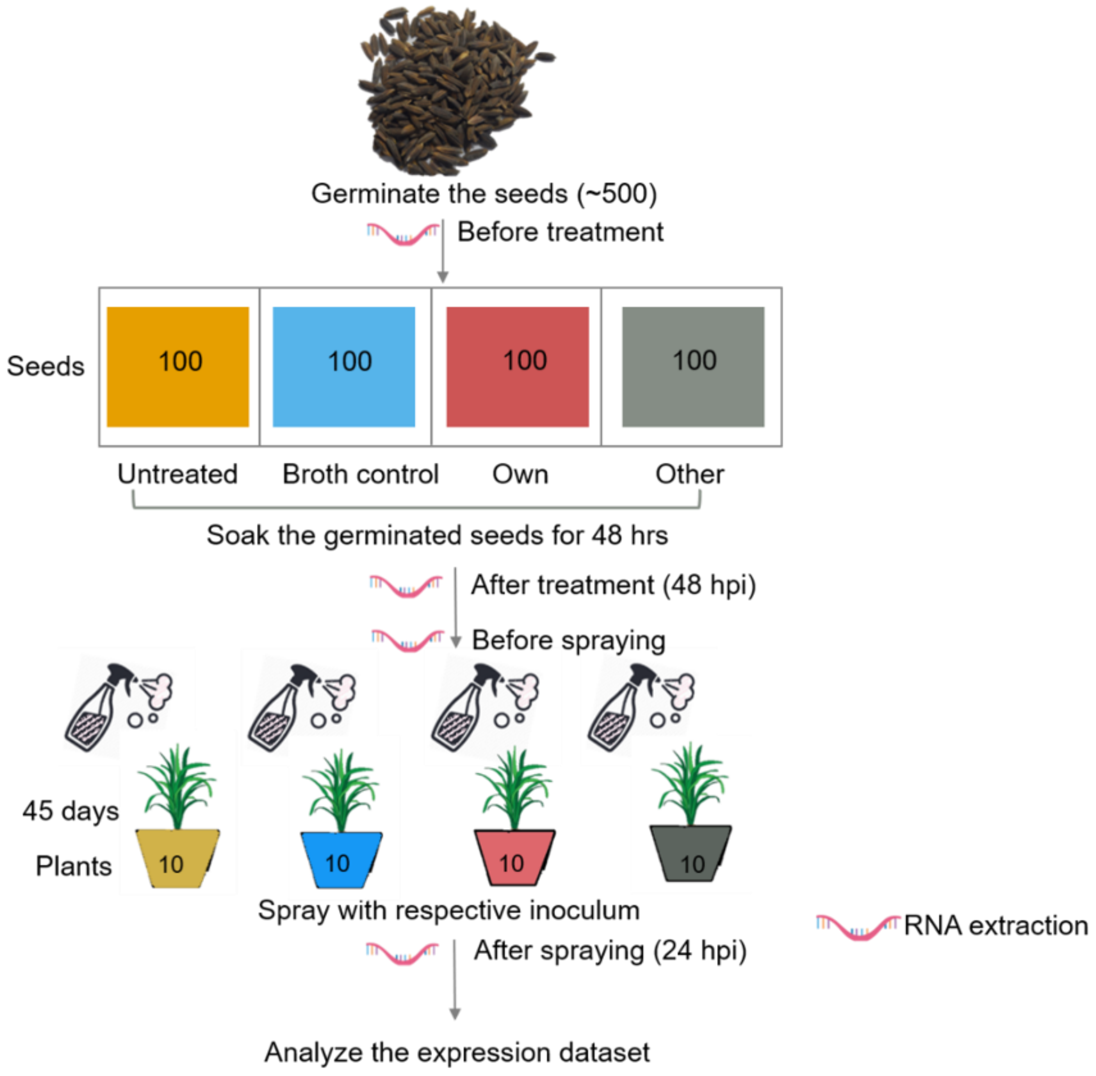

**Fig. S3**: **Change in plant height over time in field experiment**. Change in average plant height (± SD) across time (T1= 1 month, T2 = 1.5 month, T3 = 3 months after transplantation, Fig. 1) across treatments (n = 15 plants/treatment/landrace).

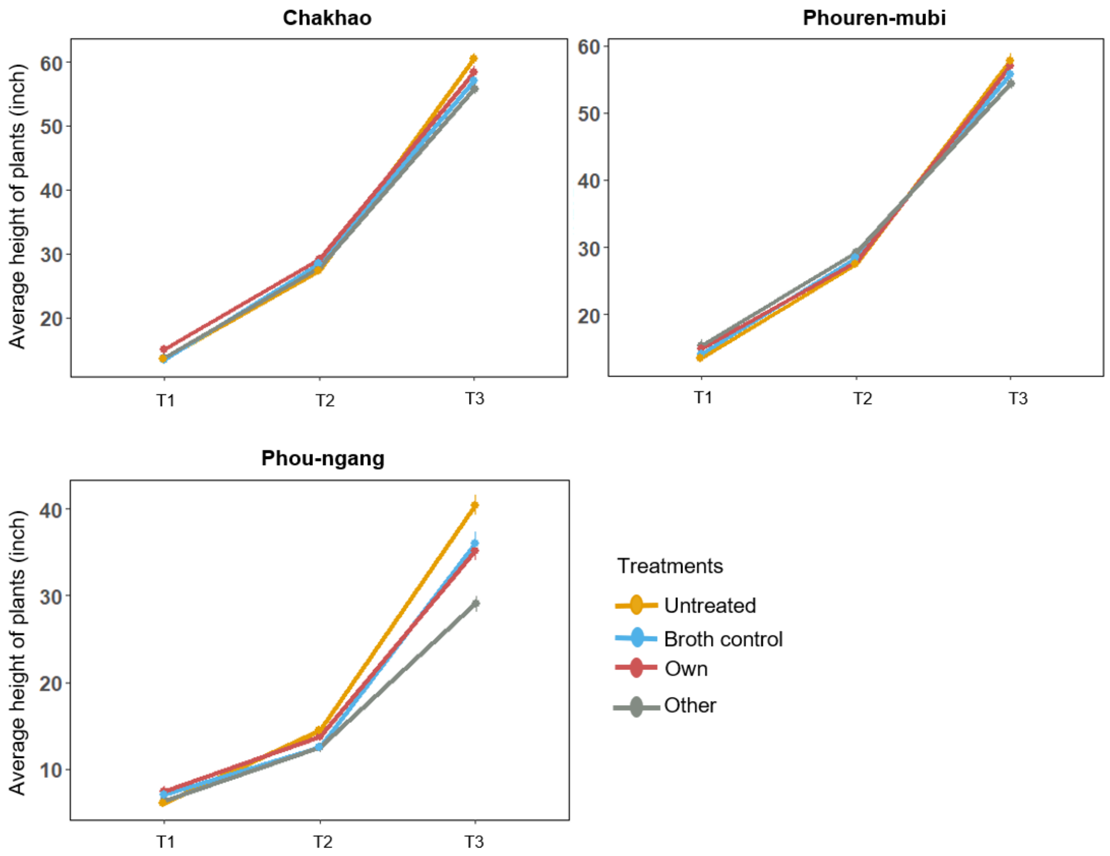

**Fig. S4**: **Plant vegetative traits as a function of treatments in the field experiment**. A. Flag leaf length; B. Total plant height (T3); C. Number of tillers; D. Number of panicles (n = 15 plants per landrace per treatment). Asterisks indicate significant pairwise differences (p<0.05), when including all replicates (grey) and when excluding influential data points (black), as estimated using GLM/ANOVA followed by Tukey's HSD.

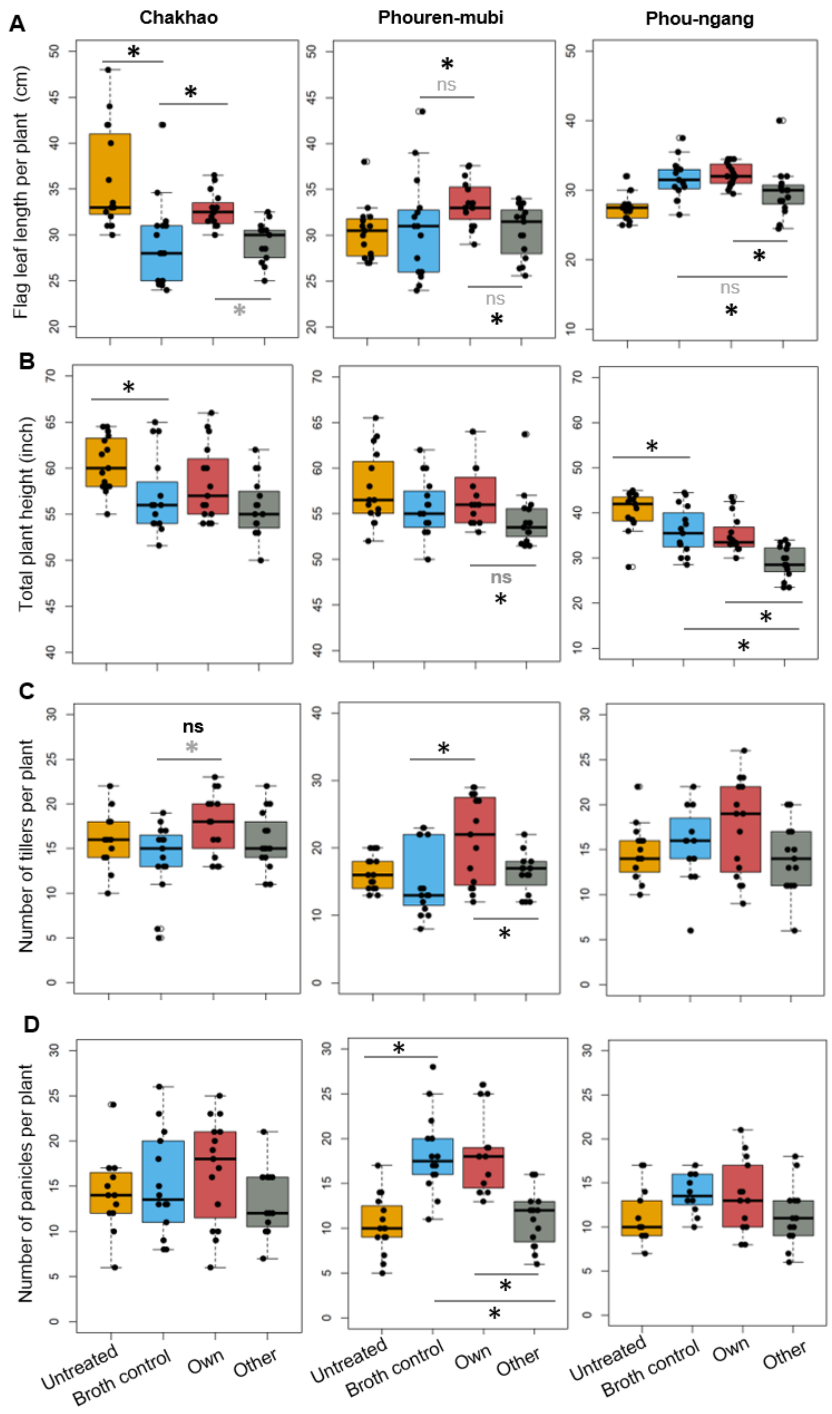

**Fig. S5**: **Plant reproductive traits as a function of treatments in the field experiment**. A. Yield (total grain weight); B. Average weight per grain; C. Number of filled grains; D. Percent filled grains (n = 12-15 plants per landrace per treatment). Asterisks indicate significant pairwise differences (p<0.05), when including all replicates (grey) and when excluding influential data points (black), as estimated using GLM/ANOVA followed by Tukey's HSD.

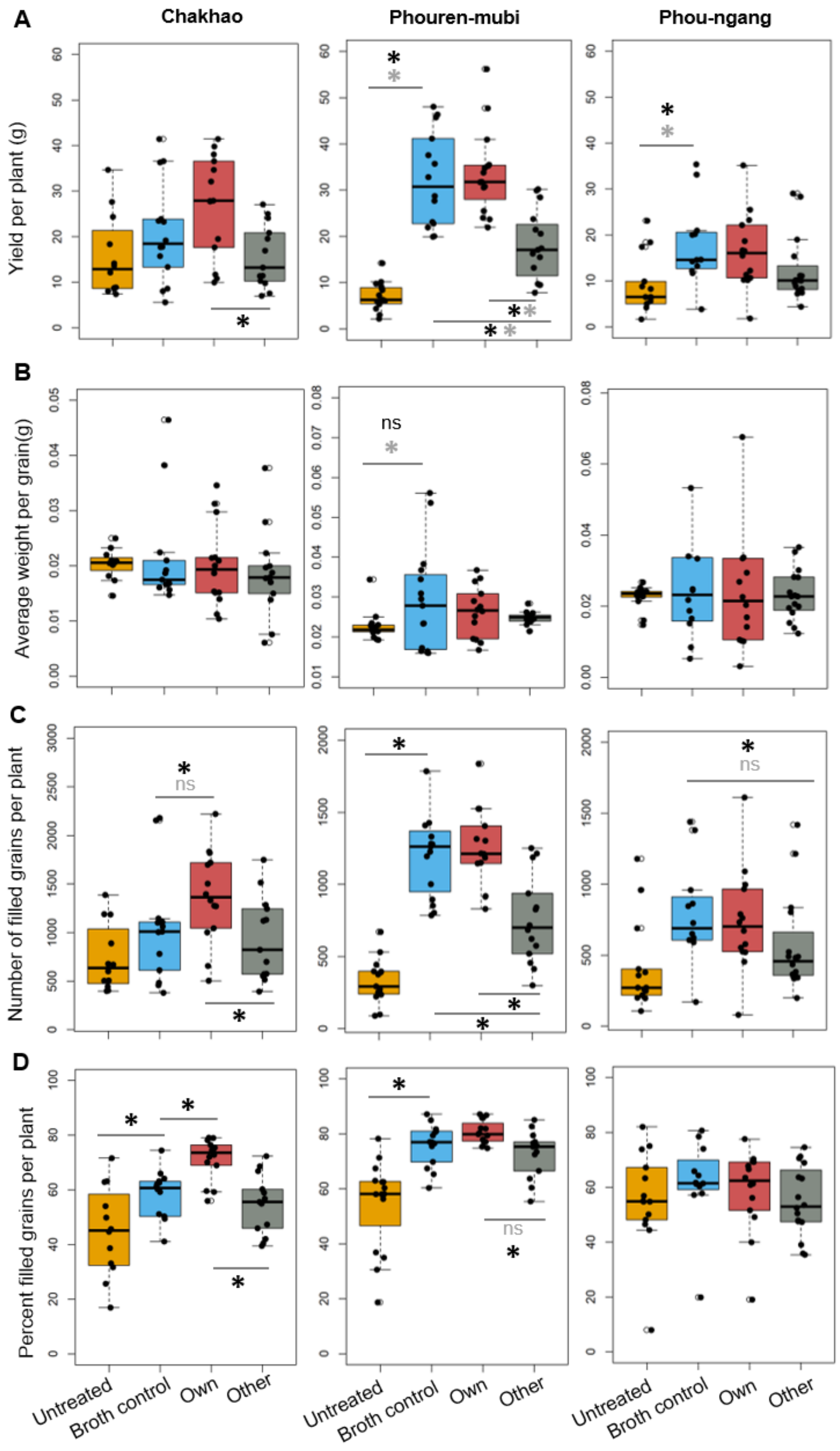

**Fig. S6**: **Colonization of plants by *Methylobacterium* in the field experiments.** Pictures show plates with leaf imprints taken after two months of foliar spraying. *Methylobacterium* colonies typically appear pink after one week of incubation. Each plate was imprinted with three leaves (from 3 different plants per treatment). From each plate, colonies with distinct morphology were chosen for 16S rRNA sequencing (Table S5).

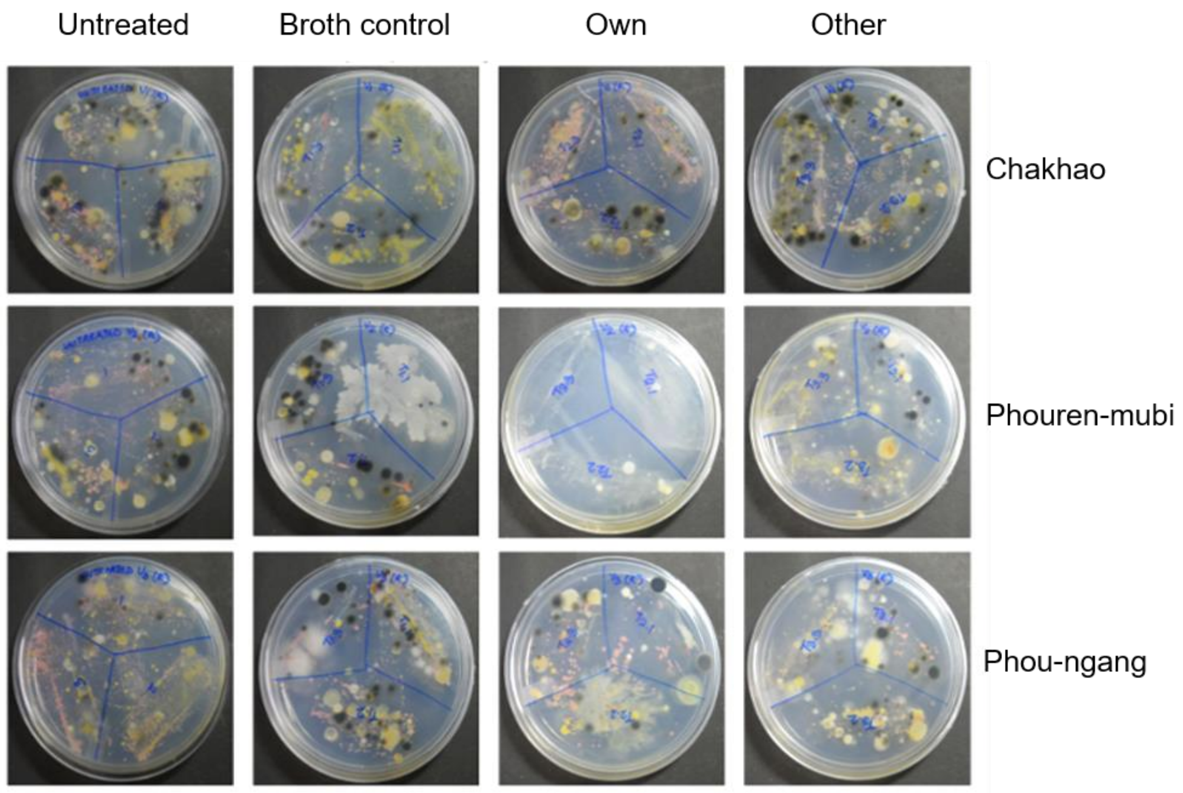

**Fig. S7**: **Phyllosphere microbiomes of experimental plants in the field**. (A) Phouren-mubi (B) Phou-ngang. Linear discriminant (LD) plots show the clustering of phyllosphere bacterial communities across treatments. Numbers in parentheses indicate biological replicates in each treatment. Axis labels indicate the proportion of variation explained and ellipsoids represent 95% confidence intervals. Boxplot show the relative abundance of own, other, and environmental *Methylobacterium* strains, as well as pathogens (see methods for details). Chi-square tests show the effect of treatment on the relative abundance of each group.

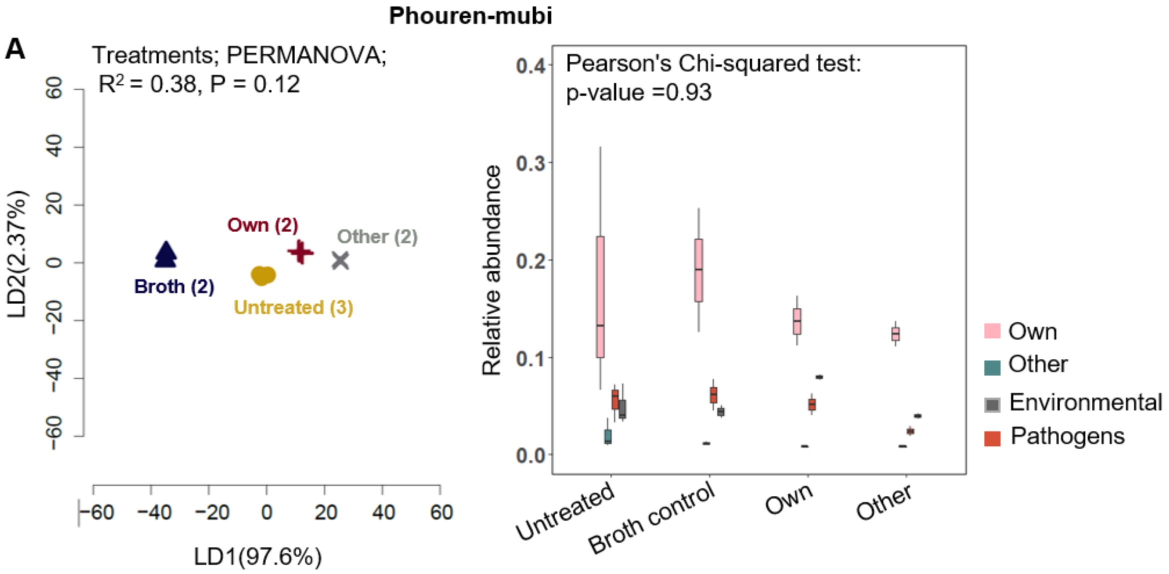


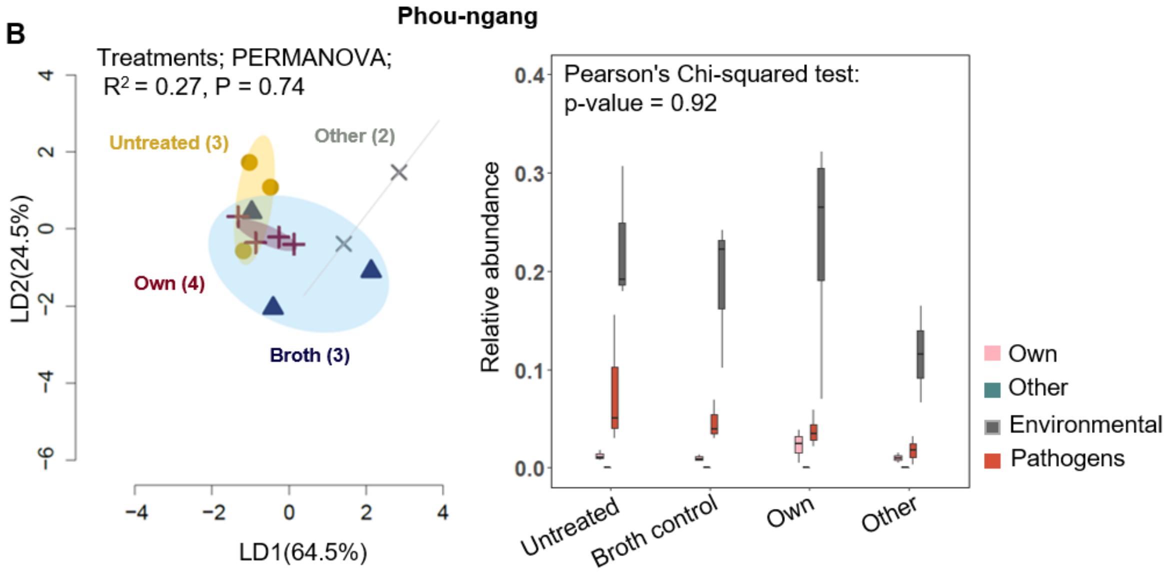

**Fig S8**: **Plant growth in the greenhouse experiment**. Plant height measured after 28th dpi (days post inoculation) in Chakhao plants (n = 10 plants/treatment). Pairwise comparisons across treatments were done using Tukey's HSD.

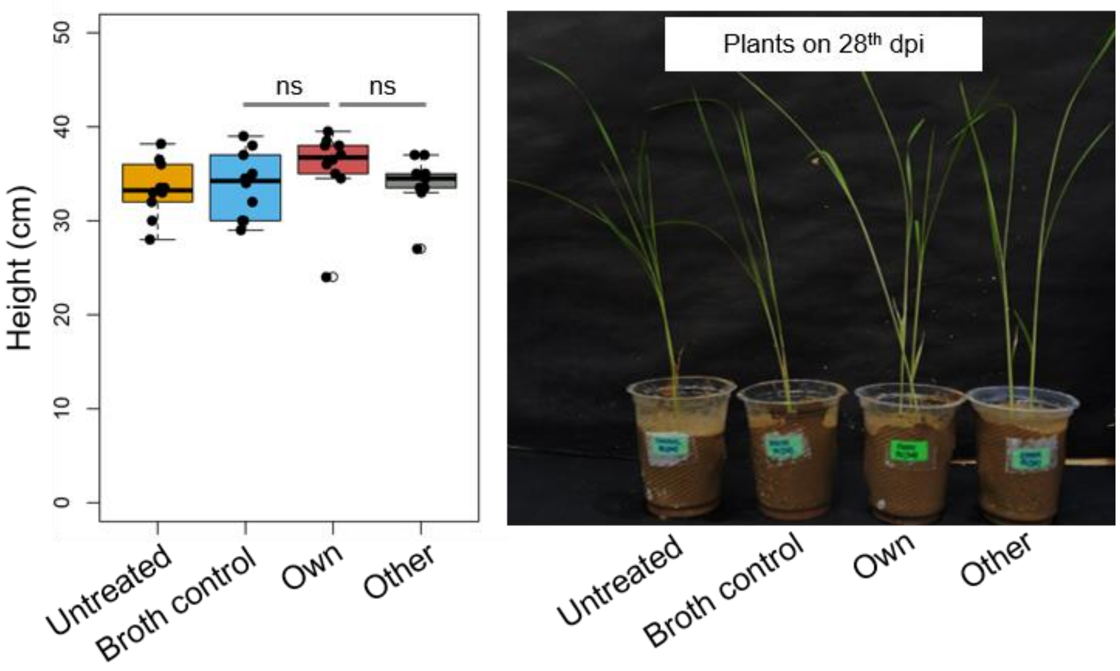

**REFERENCES**:


1. Peng H, Chen Z, Fang Z, Zhou J, Xia Z, Gao L, Chen L, Li L, Li T, Zhai W, Zhang W. 2015. Rice Xa21 primed genes and pathways that are critical for combating bacterial blight infection. Sci Rep 5:1–12.

2. Wang S, Sun Z, Wang H, Liu L, Lu F, Yang J, Zhang M, Zhang S, Guo Z, Bent AF, Sun W. 2015. Rice OsFLS2-Mediated Perception of Bacterial Flagellins Is Evaded by *Xanthomonas oryzae pvs. oryzae* and *oryzicola*. Mol Plant 8:1024–1037.

3. Mitsuhara I, Iwai T, Seo S, Yanagawa Y, Kawahigasi H, Hirose S, Ohkawa Y, Ohashi Y. 2008. Characteristic expression of twelve rice PR1 family genes in response to pathogen infection, wounding, and defense-related signal compounds (121/180). Mol Genet Genomics 279:415–427.

4. Xiao J, Cheng H, Li X, Xiao J, Xu C, Wang S. 2013. Rice WRKY13 regulates cross talk between abiotic and biotic stress signaling pathways by selective binding to different cis-elements. Plant Physiol 163:1868–1882.

5. Yim W, Seshadri S, Kim K, Lee G, Sa T. 2013. Ethylene emission and PR protein synthesis in ACC deaminase producing Methylobacterium spp. inoculated tomato plants (Lycopersicon esculentum Mill.) challenged with *Ralstonia solanacearum* under greenhouse conditions. Plant Physiol Biochem 67:95–104.

6. Solekh R, Susanto FA, Joko T, Nuringtyas TR, Purwestri YA. 2019. Phenylalanine ammonia lyase (PAL) contributes to the resistance of black rice against Xanthomonas oryzae pv. oryzae. J Plant Pathol https://doi.org/10.1007/s42161-019-00426-z.
